# 2-[1-(3-Oxo-1,3-dihydro-2-benzofuran-1-yl)-1*H*-benzimidazol-2-yl]benzoic acid methanol solvate

**DOI:** 10.1107/S1600536810026851

**Published:** 2010-07-17

**Authors:** Guang Shi, Li Ma, Hong Deng

**Affiliations:** a School of Chemistry and Environment, South China Nomal University, Guangzhou 510006, People’s Republic of China

## Abstract

The condensation of 2-carb­oxy­benzaldehyde with 1,2-phenyl­enediamine unexpectedly yielded the title compound, C_22_H_14_N_2_O_4_·CH_4_O. The benzimidazole ring system is almost perpendicular to the phthalazine ring system, making a dihedral angle of 88.4 (5)°. Inter­molecular O—H⋯N and O—H⋯O hydrogen-bonding inter­actions stabilize the crystal structure.

## Related literature

For hydrogen bonding, see: Scheiner (1997 ▶). For the role of hydrogen bonding between solvent mol­ecules and heterocyclic compounds in the formation of supra­molecules, see: Amaya & Rebek (2004 ▶); Roesky & Andruh (2003 ▶). Nelson *et al.* (1982 ▶) have reported that reaction of 2,6-diacetyl­pyridine and 1,2-phenyl­enediamine can form benzimidazole groups *via* oxidative dehydrogenation and Li *et al.* (2002 ▶) have isolated a benzimidazole derivate by the reaction of 5-bromo-2-hy­droxy­­benzaldehyde and 1,2-phenyl­enediamine in the presence of anhydrous ethanol solution. For a related structure, see: Zhang *et al.* (2009 ▶).
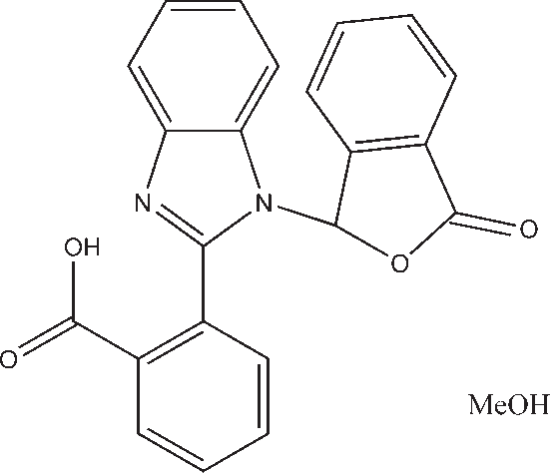


## Experimental

### 

#### Crystal data

C_22_H_14_N_2_O_4_·CH_4_O
*M* *_r_* = 402.39
Monoclinic, 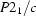

*a* = 13.7946 (8) Å
*b* = 9.7815 (7) Å
*c* = 15.3083 (9) Å
β = 103.985 (4)°
*V* = 2004.4 (2) Å^3^
*Z* = 4
Mo *K*α radiation
μ = 0.10 mm^−1^
*T* = 293 K
0.30 × 0.25 × 0.20 mm

#### Data collection

Bruker SMART APEX CCD diffractometer
Absorption correction: multi-scan (*SADABS*; Sheldrick, 1996 ▶) *T*_min_ = 0.972, *T*_max_ = 0.981
16115 measured reflections
3618 independent reflections
2220 reflections with *I* > 2σ(*I*)
*R*_int_ = 0.042

#### Refinement

*R*[*F* ^2^ > 2σ(*F* ^2^)] = 0.058
*wR*(*F* ^2^) = 0.179
*S* = 1.07
3618 reflections
273 parameters
H-atom parameters constrained
Δρ_max_ = 0.26 e Å^−3^
Δρ_min_ = −0.23 e Å^−3^

### 

Data collection: *APEX2* (Bruker, 2004 ▶); cell refinement: *SAINT* (Bruker, 2004 ▶); data reduction: *SAINT*; program(s) used to solve structure: *SHELXS97* (Sheldrick, 2008 ▶); program(s) used to refine structure: *SHELXL97* (Sheldrick, 2008 ▶); molecular graphics: *SHELXTL* (Sheldrick, 2008 ▶); software used to prepare material for publication: *SHELXTL*.

## Supplementary Material

Crystal structure: contains datablocks I, global. DOI: 10.1107/S1600536810026851/jh2172sup1.cif

Structure factors: contains datablocks I. DOI: 10.1107/S1600536810026851/jh2172Isup2.hkl

Additional supplementary materials:  crystallographic information; 3D view; checkCIF report

## Figures and Tables

**Table 1:** Hydrogen-bond geometry (Å, °)

*D*—H⋯*A*	*D*—H	H⋯*A*	*D*⋯*A*	*D*—H⋯*A*
O2—H2⋯O5	0.82	1.83	2.632 (4)	167
O5—H5*A*⋯N1^i^	0.82	1.92	2.733 (3)	173

Symmetry code: (i) 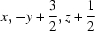.

**Fig. 1.**
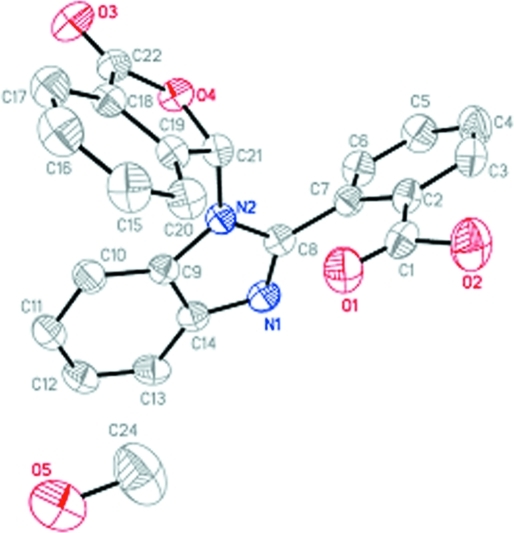
The structure of (I), showing the atomic numbering scheme. Non-H atoms are shown as 30% probability displacement ellipsoids.

**Fig. 2.**
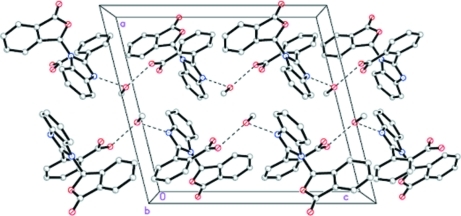
A packing view of (I) along the b axis, showing the O—H···O and O—H···N hydrogen bonds.

## supplementary crystallographic information

### Comment

Hydrogen bonding is one of an important non-covalent interaction,which plays a great role in supramolecular chemistry and material sciences (Scheiner,1997). Among solvent molecules and the hetercycle compounds the hydrogen bonding comprising O– or N– donors has been confirmed to be a useful and powerful organizing force to form supramolecules (Roesky *et al.*, 2003; Amaya *et al.*, 2004). Nelson *et al.* (Nelson *et al.*, 1982) have reported a reaction of 2,6-diacetylpyridine and 1,2-phenylenediamine can form benzimidazole groups *via* oxidative dehydrogenation and Li *et al.* (Li *et al.*, 2002) have also isolated a benzimidazole derivate in the reaction of 5-bromo-2-hydroxybenzaldehyde and 1,2-phenylenediamine in the presence of the anhydrous ethanol solution. Here, we chose 2-carboxybenzaldehyde and 1,2-phenylenediamine successfully synthesized the title compound 2-(1-(3'-phthalide-yl)-1*H*-benzoimidazol-2-yl)benzoic acid, (I).

In the main molecule of the title compound (I), (Fig. 1), the benzimidazole ring is almost perpendicular to the phthalazine ring with a dihedral angle of 88.4 (5)°, The bond lengths and angles are comparable to the similar structures (Zhang *et al.*, 2009). Intermolecular O—H···O and O—H···N interactions between the symmetry-related molecues (Table 1, Fig. 2). Adjacent molecules are stacked through  π-π interactions [*Cg*1···*Cg*2(-*x*, 1 - *y*, -*z*) = 3.578 (3) Å, where *Cg*1 and *Cg*2 are centroids of the N1/C1—C3/C8/C9 and C4—C9 rings, respectively].

### Experimental

2-carboxybenzaldehyde (0.30 g; 2 mmol) and 1,2-phenylenediamine (0.108 g; 1 mmol) were mixture in the methanol solution (30 ml), and the mixture was refluxed 3 h at 353 K. The resultant yellow precipitate was filtered and recrystallized in methanol/chloroform (4:1) solution. Standing of the solution in air at room temperation obtained colorless block crystals (I) in 79% yield.

### Refinement

water H atoms were located in a difference Fourier map and were refined isotropically, Other H-atoms on aromatic ring were placed in calculated positions with C—H = 0.93 Å; refined using a riding model with *U*_iso_(H) = 1.2 *U*_eq_(C).

### Crystal data

**Table:**

C_22_H_14_N_2_O_4_·CH_4_O	*F*(000) = 840
*M**_r_* = 402.39	*D*_x_ = 1.333 Mg m^−^^3^
Monoclinic, *P*2_1_/*c*	Mo *K*α radiation, λ = 0.71073 Å
Hall symbol: -P 2ybc	Cell parameters from 4300 reflections
*a* = 13.7946 (8) Å	θ = 2.5–25.2°
*b* = 9.7815 (7) Å	µ = 0.10 mm^−^^1^
*c* = 15.3083 (9) Å	*T* = 293 K
β = 103.985 (4)°	Block, colorless
*V* = 2004.4 (2) Å^3^	0.30 × 0.25 × 0.20 mm
*Z* = 4	

### Data collection

**Table:**

Bruker SMART APEX CCD diffractometer	3618 independent reflections
Radiation source: fine-focus sealed tube	2220 reflections with *I* > 2σ(*I*)
graphite	*R*_int_ = 0.042
ω scans	θ_max_ = 25.2°, θ_min_ = 2.5°
Absorption correction: multi-scan (*SADABS*; Sheldrick, 1996)	*h* = −16→16
*T*_min_ = 0.972, *T*_max_ = 0.981	*k* = −11→11
16115 measured reflections	*l* = −18→18

### Refinement

**Table:**

Refinement on *F*^2^	Primary atom site location: structure-invariant direct methods
Least-squares matrix: full	Secondary atom site location: difference Fourier map
*R*[*F*^2^ > 2σ(*F*^2^)] = 0.058	Hydrogen site location: inferred from neighbouring sites
*wR*(*F*^2^) = 0.179	H-atom parameters constrained
*S* = 1.07	*w* = 1/[σ^2^(*F*_o_^2^) + (0.0793*P*)^2^ + 0.7223*P*] where *P* = (*F*_o_^2^ + 2*F*_c_^2^)/3
3618 reflections	(Δ/σ)_max_ = 0.005
273 parameters	Δρ_max_ = 0.26 e Å^−^^3^
0 restraints	Δρ_min_ = −0.23 e Å^−^^3^

### Special details

**Table:**

Geometry. All e.s.d.'s (except the e.s.d. in the dihedral angle between two l.s. planes) are estimated using the full covariance matrix. The cell e.s.d.'s are taken into account individually in the estimation of e.s.d.'s in distances, angles and torsion angles; correlations between e.s.d.'s in cell parameters are only used when they are defined by crystal symmetry. An approximate (isotropic) treatment of cell e.s.d.'s is used for estimating e.s.d.'s involving l.s. planes.
Refinement. Refinement of *F*^2^ against ALL reflections. The weighted *R*-factor *wR* and goodness of fit *S* are based on *F*^2^, conventional *R*-factors *R* are based on *F*, with *F* set to zero for negative *F*^2^. The threshold expression of *F*^2^ > σ(*F*^2^) is used only for calculating *R*-factors(gt) *etc*. and is not relevant to the choice of reflections for refinement. *R*-factors based on *F*^2^ are statistically about twice as large as those based on *F*, and *R*- factors based on ALL data will be even larger.

### Fractional atomic coordinates and isotropic or equivalent isotropic displacement parameters (Å^2^)

**Table:**

	*x*	*y*	*z*	*U*_iso_*/*U*_eq_	
C1	0.2698 (3)	0.7568 (4)	0.3140 (2)	0.0646 (8)	
C2	0.2114 (2)	0.7749 (3)	0.2192 (2)	0.0574 (8)	
C3	0.1577 (3)	0.8940 (3)	0.1918 (2)	0.0735 (9)	
H3	0.1539	0.9603	0.2344	0.088*	
C4	0.1102 (3)	0.9157 (4)	0.1035 (3)	0.0837 (11)	
H4	0.0743	0.9960	0.0869	0.100*	
C5	0.1153 (3)	0.8195 (4)	0.0393 (2)	0.0802 (10)	
H5	0.0842	0.8355	−0.0208	0.096*	
C6	0.1665 (2)	0.6995 (3)	0.0642 (2)	0.0675 (9)	
H6	0.1692	0.6343	0.0207	0.081*	
C7	0.2148 (2)	0.6743 (3)	0.15465 (19)	0.0534 (7)	
C8	0.2695 (2)	0.5440 (3)	0.17289 (17)	0.0514 (7)	
C9	0.3092 (2)	0.3309 (3)	0.21960 (18)	0.0504 (7)	
C10	0.3180 (2)	0.1996 (3)	0.2542 (2)	0.0619 (8)	
H10	0.2747	0.1664	0.2872	0.074*	
C11	0.3938 (2)	0.1202 (4)	0.2373 (2)	0.0748 (10)	
H11	0.4015	0.0313	0.2593	0.090*	
C12	0.4587 (3)	0.1686 (4)	0.1886 (3)	0.0774 (10)	
H12	0.5088	0.1113	0.1786	0.093*	
C13	0.4514 (2)	0.2990 (4)	0.1547 (2)	0.0700 (9)	
H13	0.4955	0.3313	0.1221	0.084*	
C14	0.3749 (2)	0.3812 (3)	0.17105 (19)	0.0550 (7)	
C15	0.2117 (3)	0.3034 (4)	0.4943 (2)	0.0780 (10)	
H15	0.2504	0.3200	0.5520	0.094*	
C16	0.1467 (3)	0.1954 (4)	0.4806 (2)	0.0805 (11)	
H16	0.1423	0.1408	0.5293	0.097*	
C17	0.0878 (3)	0.1656 (4)	0.3965 (2)	0.0698 (9)	
H17	0.0444	0.0913	0.3868	0.084*	
C18	0.0965 (2)	0.2521 (3)	0.32720 (18)	0.0519 (7)	
C19	0.1618 (2)	0.3603 (3)	0.34079 (17)	0.0499 (7)	
C20	0.2215 (2)	0.3885 (3)	0.42476 (19)	0.0666 (9)	
H20	0.2662	0.4613	0.4343	0.080*	
C21	0.1534 (2)	0.4328 (3)	0.25260 (17)	0.0498 (7)	
H21	0.1296	0.5263	0.2572	0.060*	
C22	0.0433 (2)	0.2488 (3)	0.2319 (2)	0.0546 (7)	
C24	0.4396 (5)	0.6942 (6)	0.5552 (4)	0.149 (2)	
H24A	0.5088	0.7002	0.5865	0.223*	
H24B	0.4319	0.6279	0.5078	0.223*	
H24C	0.4008	0.6671	0.5966	0.223*	
N1	0.34805 (17)	0.5147 (3)	0.14283 (15)	0.0571 (7)	
N2	0.24148 (16)	0.4369 (2)	0.22025 (14)	0.0483 (6)	
O1	0.3086 (2)	0.6524 (3)	0.34359 (16)	0.0873 (8)	
O2	0.2764 (2)	0.8683 (3)	0.36180 (18)	0.1010 (9)	
H2	0.3091	0.8530	0.4131	0.151*	
O3	−0.01827 (18)	0.1711 (3)	0.19162 (15)	0.0789 (7)	
O4	0.07646 (14)	0.3553 (2)	0.18969 (12)	0.0556 (5)	
O5	0.4054 (2)	0.8271 (3)	0.51697 (18)	0.1090 (10)	
H5A	0.3930	0.8763	0.5563	0.163*	

### Atomic displacement parameters (Å^2^)

**Table:**

	*U*^11^	*U*^22^	*U*^33^	*U*^12^	*U*^13^	*U*^23^
C1	0.073 (2)	0.058 (2)	0.063 (2)	−0.0139 (17)	0.0172 (17)	−0.0095 (17)
C2	0.0678 (19)	0.0495 (18)	0.0555 (18)	−0.0065 (15)	0.0161 (15)	0.0008 (14)
C3	0.087 (2)	0.055 (2)	0.079 (2)	−0.0016 (18)	0.0216 (19)	0.0016 (17)
C4	0.096 (3)	0.062 (2)	0.088 (3)	0.009 (2)	0.013 (2)	0.016 (2)
C5	0.088 (3)	0.079 (3)	0.065 (2)	0.001 (2)	0.0033 (18)	0.019 (2)
C6	0.081 (2)	0.067 (2)	0.0524 (18)	−0.0029 (18)	0.0126 (16)	0.0050 (16)
C7	0.0568 (17)	0.0519 (17)	0.0526 (17)	−0.0054 (14)	0.0155 (14)	0.0049 (14)
C8	0.0548 (17)	0.0563 (18)	0.0424 (15)	−0.0016 (14)	0.0108 (13)	−0.0001 (13)
C9	0.0497 (16)	0.0541 (17)	0.0456 (15)	0.0014 (13)	0.0084 (13)	−0.0025 (13)
C10	0.0607 (19)	0.0605 (19)	0.066 (2)	0.0062 (15)	0.0183 (15)	0.0060 (16)
C11	0.067 (2)	0.066 (2)	0.091 (3)	0.0134 (18)	0.0187 (19)	0.0115 (19)
C12	0.062 (2)	0.078 (3)	0.094 (3)	0.0177 (18)	0.0219 (19)	0.001 (2)
C13	0.0529 (18)	0.086 (3)	0.074 (2)	0.0035 (17)	0.0220 (16)	−0.0046 (19)
C14	0.0536 (17)	0.0602 (19)	0.0504 (16)	−0.0026 (14)	0.0111 (14)	−0.0015 (14)
C15	0.100 (3)	0.082 (2)	0.0461 (18)	0.004 (2)	0.0067 (17)	0.0040 (18)
C16	0.106 (3)	0.089 (3)	0.0502 (19)	0.011 (2)	0.0255 (19)	0.0221 (19)
C17	0.081 (2)	0.073 (2)	0.061 (2)	0.0001 (18)	0.0259 (18)	0.0145 (17)
C18	0.0551 (17)	0.0579 (18)	0.0463 (16)	0.0042 (14)	0.0194 (13)	0.0032 (13)
C19	0.0565 (17)	0.0525 (17)	0.0423 (15)	0.0079 (14)	0.0151 (13)	0.0023 (13)
C20	0.077 (2)	0.070 (2)	0.0497 (18)	0.0010 (17)	0.0080 (15)	−0.0033 (16)
C21	0.0567 (17)	0.0496 (16)	0.0437 (15)	0.0018 (13)	0.0136 (13)	0.0005 (13)
C22	0.0547 (17)	0.0605 (19)	0.0508 (17)	−0.0030 (15)	0.0167 (14)	0.0003 (15)
C24	0.196 (6)	0.126 (4)	0.107 (4)	0.046 (4)	0.003 (4)	−0.023 (3)
N1	0.0575 (15)	0.0634 (16)	0.0525 (14)	−0.0064 (12)	0.0172 (12)	0.0006 (12)
N2	0.0515 (13)	0.0486 (13)	0.0471 (13)	0.0029 (11)	0.0163 (10)	0.0039 (11)
O1	0.1053 (19)	0.0746 (17)	0.0682 (15)	0.0027 (15)	−0.0062 (13)	−0.0027 (13)
O2	0.138 (2)	0.0844 (18)	0.0751 (17)	0.0012 (17)	0.0144 (16)	−0.0227 (14)
O3	0.0792 (15)	0.0920 (18)	0.0642 (14)	−0.0309 (14)	0.0147 (12)	−0.0024 (13)
O4	0.0561 (12)	0.0671 (13)	0.0422 (10)	−0.0067 (10)	0.0089 (9)	0.0045 (9)
O5	0.117 (2)	0.138 (3)	0.0689 (16)	0.024 (2)	0.0179 (16)	−0.0279 (17)

### Geometric parameters (Å, °)

**Table:**

C1—O1	1.190 (4)	C13—H13	0.9300
C1—O2	1.305 (4)	C14—N1	1.397 (4)
C1—C2	1.489 (4)	C15—C16	1.368 (5)
C2—C3	1.390 (4)	C15—C20	1.383 (5)
C2—C7	1.404 (4)	C15—H15	0.9300
C3—C4	1.369 (5)	C16—C17	1.378 (5)
C3—H3	0.9300	C16—H16	0.9300
C4—C5	1.375 (5)	C17—C18	1.385 (4)
C4—H4	0.9300	C17—H17	0.9300
C5—C6	1.376 (5)	C18—C19	1.372 (4)
C5—H5	0.9300	C18—C22	1.467 (4)
C6—C7	1.405 (4)	C19—C20	1.377 (4)
C6—H6	0.9300	C19—C21	1.505 (4)
C7—C8	1.473 (4)	C20—H20	0.9300
C8—N1	1.307 (3)	C21—N2	1.419 (3)
C8—N2	1.381 (3)	C21—O4	1.461 (3)
C9—C10	1.384 (4)	C21—H21	0.9800
C9—C14	1.393 (4)	C22—O3	1.194 (3)
C9—N2	1.397 (3)	C22—O4	1.362 (3)
C10—C11	1.376 (4)	C24—O5	1.456 (6)
C10—H10	0.9300	C24—H24A	0.9600
C11—C12	1.380 (5)	C24—H24B	0.9600
C11—H11	0.9300	C24—H24C	0.9600
C12—C13	1.372 (5)	O2—H2	0.8200
C12—H12	0.9300	O5—H5A	0.8200
C13—C14	1.397 (4)		
			
O1—C1—O2	122.6 (3)	C13—C14—N1	129.6 (3)
O1—C1—C2	124.1 (3)	C16—C15—C20	122.0 (3)
O2—C1—C2	113.3 (3)	C16—C15—H15	119.0
C3—C2—C7	118.7 (3)	C20—C15—H15	119.0
C3—C2—C1	121.1 (3)	C15—C16—C17	121.5 (3)
C7—C2—C1	120.0 (3)	C15—C16—H16	119.2
C4—C3—C2	121.4 (3)	C17—C16—H16	119.2
C4—C3—H3	119.3	C16—C17—C18	116.4 (3)
C2—C3—H3	119.3	C16—C17—H17	121.8
C3—C4—C5	120.3 (3)	C18—C17—H17	121.8
C3—C4—H4	119.8	C19—C18—C17	122.1 (3)
C5—C4—H4	119.8	C19—C18—C22	108.7 (2)
C6—C5—C4	119.8 (3)	C17—C18—C22	129.3 (3)
C6—C5—H5	120.1	C18—C19—C20	121.2 (3)
C4—C5—H5	120.1	C18—C19—C21	108.8 (2)
C5—C6—C7	120.9 (3)	C20—C19—C21	130.0 (3)
C5—C6—H6	119.6	C19—C20—C15	116.7 (3)
C7—C6—H6	119.6	C19—C20—H20	121.6
C2—C7—C6	118.8 (3)	C15—C20—H20	121.6
C2—C7—C8	125.1 (3)	N2—C21—O4	109.3 (2)
C6—C7—C8	116.0 (3)	N2—C21—C19	116.1 (2)
N1—C8—N2	112.3 (2)	O4—C21—C19	103.4 (2)
N1—C8—C7	123.6 (2)	N2—C21—H21	109.2
N2—C8—C7	124.1 (2)	O4—C21—H21	109.2
C10—C9—C14	121.6 (3)	C19—C21—H21	109.2
C10—C9—N2	133.1 (3)	O3—C22—O4	121.4 (3)
C14—C9—N2	105.3 (2)	O3—C22—C18	130.6 (3)
C11—C10—C9	116.9 (3)	O4—C22—C18	108.0 (2)
C11—C10—H10	121.5	O5—C24—H24A	109.5
C9—C10—H10	121.5	O5—C24—H24B	109.5
C10—C11—C12	122.0 (3)	H24A—C24—H24B	109.5
C10—C11—H11	119.0	O5—C24—H24C	109.5
C12—C11—H11	119.0	H24A—C24—H24C	109.5
C13—C12—C11	121.7 (3)	H24B—C24—H24C	109.5
C13—C12—H12	119.2	C8—N1—C14	106.0 (2)
C11—C12—H12	119.2	C8—N2—C9	106.6 (2)
C12—C13—C14	117.2 (3)	C8—N2—C21	125.2 (2)
C12—C13—H13	121.4	C9—N2—C21	127.7 (2)
C14—C13—H13	121.4	C1—O2—H2	109.5
C9—C14—C13	120.7 (3)	C22—O4—C21	111.0 (2)
C9—C14—N1	109.7 (2)	C24—O5—H5A	109.5

### Hydrogen-bond geometry (Å, °)

**Table:**

*D*—H···*A*	*D*—H	H···*A*	*D*···*A*	*D*—H···*A*
O2—H2···O5	0.82	1.83	2.632 (4)	167
O5—H5A···N1^i^	0.82	1.92	2.733 (3)	173

Symmetry codes: (i) *x*, −*y*+3/2, *z*+1/2.
